# A Low-Carbohydrate Ketogenic Diet and Treadmill Training Enhanced Fatty Acid Oxidation Capacity but Did Not Enhance Maximal Exercise Capacity in Mice

**DOI:** 10.3390/nu13020611

**Published:** 2021-02-13

**Authors:** Sihui Ma, Jiao Yang, Takaki Tominaga, Chunhong Liu, Katsuhiko Suzuki

**Affiliations:** 1 Faculty of Sport Sciences, Waseda University, Tokorozawa 3591192, Japan; masihui@toki.waseda.jp; 2 Japan Society for the Promotion of Sciences, Chiyoda-ku, Tokyo 1020083, Japan; takaki.k-bbc@akane.waseda.jp; 3 Graduate School of Sport Sciences, Waseda University, Tokorozawa 3591192, Japan; yangjiao_626@163.com; 4 College of Food Sciences, South China Agricultural University, Guangzhou 510642, China; 5 Guangdong Provincial Key Laboratory of Food Quality and Safety, Guangzhou 510642, China

**Keywords:** ketogenic diet, exercise capacity, fatty acid oxidation, low-carbohydrate diet

## Abstract

The low-carbohydrate ketogenic diet (LCKD) is a dietary approach characterized by the intake of high amounts of fat, a balanced amount of protein, and low carbohydrates, which is insufficient for metabolic demands. Previous studies have shown that an LCKD alone may contribute to fatty acid oxidation capacity, along with endurance. In the present study, we combined a 10-week LCKD with an 8-week forced treadmill running program to determine whether training in conjunction with LCKD enhanced fatty acid oxidation capacity, as well as whether the maximal exercise capacity would be affected by an LCKD or training in a mice model. We found that the lipid pool and fatty acid oxidation capacity were both enhanced following the 10-week LCKD. Further, key fatty acid oxidation related genes were upregulated. In contrast, the 8-week training regimen had no effect on fatty acid and ketone body oxidation. Key genes involved in carbohydrate utilization were downregulated in the LCKD groups. However, the improved fatty acid oxidation capacity did not translate into an enhanced maximal exercise capacity. In summary, while favoring the fatty acid oxidation system, an LCKD, alone or combined with training, had no beneficial effects in our intensive exercise-evaluation model. Therefore, an LCKD may be promising to improve endurance in low- to moderate-intensity exercise, and may not be an optimal choice for those partaking in high-intensity exercise.

## 1. Introduction

Nutrition relates to the idea of developing a balanced diet for mankind [1]. In fact, few topics have caused as much controversy as the so-called “best diet” debate. Today, even when people can freely choose what to put on their plates, a truly optimal choice remains elusive. With an ever-increasing number of studies in the fields of human nutrition and dietetics, as well as the explosive spread of information, there is no shortage of information on diets in the current day.

Simply put, the main questions within nutrition that are yet to be conclusively answered are: 1. How to eat? 2. When to eat? 3. What to eat? The answer to question 1 seems relatively simple, but only to a certain extent, as the notion that “quality counts more than calories” advises us to choose fewer refined and processed foods, while trying to expand and diversify our food plate [2]. Regarding question 2, intermittent fasting (including Ramadan intermittent fasting and 16/8 time-restricted feeding) has exhibited preferred metabolic effects according to recent studies [3]. Further, various chrono-nutrition studies advise us to eat in compliance with our circadian clock [4].

However, when it comes to “what to eat,” despite various eye-dazzling diet recommendations and novel direct-energy sources (e.g., ketone drinks) entering the market, carbohydrates (starch and sugars), fat, and protein remain the major macronutrients [5]. In the past decades, diets with different macronutrient bases were compared, resulting in a heated dispute over what the best approach is. From the very-low-carbohydrate “Atkins” diet to the high-fat “ketogenic” diet, from the high-fiber, high-unsaturated-fat “Paleo” diet to the low-protein high-glycemic diets, a multitude of possibilities for dietary macronutrient reconstitution have been discussed [6,7]. While we can be certain that a “one-size-fits-all” diet does not exist, a comprehensive comparison of representative diets is still missing.

The low-carbohydrate ketogenic diet (LCKD) is a nutritional approach characterized by the intake of high amounts of fat, a balanced amount of protein, and low carbohydrates, which is insufficient for metabolic demands [8]. The LCKD was initially used for the treatment for epilepsy, but studies have shown that it may have a variety of clinically relevant effects, with implications for weight control, diabetes, pain and inflammation, and cancer. Apart from its clinical use, the LCKD also has potential as a nutrition approach for common individuals and trained athletes alike [9]. In the classic ketogenic diet (KD), glucose-induced metabolic activity should account for less than 5% of the daily calorie intake [10,11]. A low carbohydrate intake fails to maintain oxaloacetate required for the Krebs cycle, pushing the body to adopt a fat-centered metabolism. Ketogenesis is the process of fat adaptation, during which fatty acids are converted into acetyl-CoA via β-oxidation within the mitochondria, followed by conversion into ketone bodies (KBs). KBs include acetoacetate (AcAc), 3-hydroxybutyrate (3-HB), and acetone, and are the main energy source during ketosis, the term used to define this metabolic state. Adaptation to KB utilization can liberate more available energy than glucose adaptation.

Both adipose tissue and muscle tissue are currently regarded as endocrine organs. Crosstalks between white adipose tissue, brown adipose tissue, and muscle tissue, through production and secretion of adipokines and myokines, play crucial roles in energy metabolism and endurance [12]. For instance, adiponectin, an adipokine secreted by adipocytes, is involved in glucose-level regulation and alleviation of inflammation. Interleukin (IL)-6, a myokine produced by skeletal muscle, is considered to play key roles in mediating fatty acid oxidation [13]. Although the LCKD has an undeniable effect on weight control, its effect on metabolic responses, especially on crosstalk between tissues, has remained unclear [14].

Fibroblast growth factor 21 (FGF21) is a key regulator of various physiological changes in response to environmental stress. While consuming a LCKD, FGF21 might be elevated, and thus contribute to energy homeostasis and promote weight loss [15]. Hormones, including testosterone and insulin-like growth factor 1 (IGF-1) may contribute to the maintenance of muscle strength. Testosterone administration to elderly men was reported to increase muscle strength, and this increase might be attributed to the stimulation of the intramuscular IGF-1 system [16]. A recent study showed that a 45-day isocaloric, very-low-calorie ketogenic diet incorporating whey protein may be more effective in maintaining muscle performance in obese patients, while circulating IGF-1 may contribute to the maintaining effect [17]. However, it remains unclear how the hormones alter during an LCKD combined with training.

In previous studies, it was reported that an 8-week LCKD induced favorable changes in lipolytic and ketolytic gene expression, thus improving exhaustive exercise capacity [18,19]. It has been established that endurance training enhances the fatty acid oxidation system. Therefore, whether training in combination with a ketogenic diet can further enhance exercise capacity has emerged as a new relevant question. To address it, we designed a 10-week LCKD feeding study with an 8-week treadmill training regimen, focusing on multiorgan changes in metabolism-associated gene expression and plasma biomarkers, as well as broader physiological changes.

## 2. Materials and Methods

### 2.1. Mice and Diets

We designed two diets: a chow diet (control: Con) and a high-fat, low-carbohydrate ketogenic diet (LCKD). Their nutritional information is shown in Table 1. Both diets were obtained from TROPHIC Animal Feed High-Tech Co., Ltd. (Nantong, Jiangsu, China).

Male C57BL/6J mice (*n* = 32) were purchased from Takasugi Experimental Animals Supply (Kasukabe, Japan) at 8 weeks of age, and were allowed to adapt to the environment for a week before formal experimentation commenced. Four animals were housed together in a cage (27 × 17 × 13 cm) under a controlled environment with a 12-h light–dark cycle (lights on at 08:00 and off at 20:00). The experimental procedures were approved and followed the Guiding Principles for the Care and Use of Animals of the Academic Research Ethical Review Committee of Waseda University (2018-A114). All mice were randomly divided into four groups: chow diet (Con), including chow diet with promotion of sedentary behavior (*n* = 8) and chow diet plus training (Con + T, *n* = 8, T is the abbreviation for training), as well as an LCKD with promotion of sedentary behavior (*n* = 8) and an LCKD plus exercise (LCKD + T, *n* = 8). Mice on all diets had access to food ad libitum for 10 weeks, starting at 9 weeks of age.

### 2.2. Training Procedures, Forelimb Grip Strength Test, and Maximal Workload

All mice in the training group performed 1 h of forced treadmill running every training day. Training was done 5 days/week. The running speed was set to 22.5 m/min. The treadmill (Natsume, Tokyo, Japan) was set to a five-grade incline. The training period lasted 8 weeks.

A grip-strength meter (GPM-100; Melquest, Toyama, Japan) was used to measure forelimb grip strength. As a mouse grasped the bar, the peak pull force in grams was recorded on a digital force transducer. In the conventional version of this test, the mouse is allowed to grasp the bar mounted on the force gauge. The gauge is reset to 0 g after stabilization, and the tail of the mouse is slowly pulled back by the inspector. Tension is recorded based on the gauge at the time the mouse released its forepaws from the bar. For the modified version of this test used in the current study, the gauge was rotated vertically and fixed to the metal stand to keep the system immobilized. The measurement procedure was identical to that in the conventional test, except for the direction in which the tail of the mouse was pulled by the inspector. For each test, trials in which only one forepaw or the hindlimbs were used, as well as those in which the mouse turned during the pull or left the bar without resistance, were excluded. Given that the speed of the tail pull can influence the measurement, we conducted the procedure at a constant speed that was sufficiently slow to permit mice to build up resistance against it. We performed three consecutive measurements per test at 30-second intervals.

Maximal exercise capacity was evaluated using a treadmill, according to a previously described method [20,21]. One week before training began, all mice were familiarized with running on a motorized rodent treadmill, as previously described [14]. After familiarization, mice performed two graded exercise performance tests at two time points, the first at 3 days before training, and the second 3 days after the 8-week training period. Tests started at 9 m/min for 9 min, going up to 10 m/min, and increasing by 2.5 m/min every 3 min. The starting incline was 0 and was raised by 5 every 9 min, with a maximal incline of 15. Exhaustion was defined as the inability of mice to continue regular treadmill running despite stimulation by repeated tapping on the back. At this point, running time (in min) was recorded, and each mouse was removed from the treadmill and returned to its home cage. Exercise capacity was expressed in time (min) and work (kg·m). The work performed (kg·m) was calculated as the product of body weight (kg) and vertical distance (m), where:

vertical distance = (distance run) (sin θ)(1)

where θ was the angle of the treadmill, from 0 to 15 [15,16]. A second pair of exercise performance tests was completed after the training period, and changes in exercise capacity were calculated.

### 2.3. Sampling and Plasma Biochemical Assessment

Two days after the final training, mice were fasted for 12 h and sacrificed under light anesthesia with isoflurane (Abbott, Tokyo, Japan). Blood samples were taken from the abdominal aorta using heparin under inhalant isoflurane-induced mild anesthesia. Tissues and organs were immediately excised and frozen in liquid nitrogen. Plasma was obtained from blood samples by centrifugation at 1500× *g* for 10 min at 4 °C. These samples were stored at −80 °C until analysis. The whole liver, epididymal fat, brown fat, and a muscle fascicle from the left hind leg, containing the gastrocnemius, soleus, and plantaris muscles, were weighed. Plasma levels of glucose, non-esterified fatty acids (NEFA), triglyceride (TG), lipase, amylase, aspartate transaminase (AST), creatine kinase (CK), lactate dehydrogenase (LDH), blood urea nitrogen (BUN), urea acid (UA), creatinine, total cholesterol (T-CHO), high-density lipoprotein cholesterol (H-CHO), low-density lipoprotein cholesterol (L-CHO), and albumin were measured by Koutou-Biken Co. (Tsukuba, Japan). A commercial assay kit was employed to measure the β-hydroxybutyrate concentration (Cayman, MI, USA).

### 2.4. Real-Time PCR

Total RNA was extracted from the gastrocnemius muscle (fast-twitch muscle) and soleus muscle (slow-twitch muscle) using the RNeasy Fibrous Mini Kit, as well as from epididymal adipose tissue and brown adipose tissue using the RNeasy Lipid Tissue Mini Kit (Qiagen, Valencia, CA, USA), according to the manufacturer’s instructions. The purity and concentration of total RNA were assessed using the NanoDrop system (NanoDrop Technologies, Wilmington, DE, USA). Total RNA was reverse-transcribed to cDNA using the High Capacity cDNA Reverse Transcription Kit (Applied Biosystems, Foster City, CA, USA) according to the manufacturer’s instructions. PCR was performed on the Fast 7500 real-time PCR system (Applied Biosystems) using the Fast SYBR^®^ Green PCR Master Mix (Applied Biosystems). The thermal cycle profiles consisted of 10 min at 95 °C for denaturation, followed by 40 cycles at 95 °C for 3 s and annealing at 60 °C for 15 s. 18s ribosomal RNA was used as the housekeeping gene, and the ⊿⊿CT method was used to quantify target gene expression. All data are represented as fold change relative to expression values of the Con group. Primers used for real-time PCR are shown in Table 2. 

### 2.5. Plasma Fibroblast Growth Factor 21 (FGF21) and Testosterone Assessment

Plasma FGF21 concentrations were measured using the R&D Mouse FGF21 ELISA kit (R&D Systems, Minneapolis, MN, USA) according to the manufacturer’s instructions. Plasma testosterone concentrations were measured using a commercial ELISA kit (Cayman, MI, USA) according to the manufacturer’s instructions.

### 2.6. Muscle Glycogen Assessment

Gastrocnemius muscle glycogen content was measured using a commercial kit (Cayman, MI, USA) according to the manufacturer’s instructions.

### 2.7. Statistical Analysis

Data are presented as the mean ± standard deviation (SD). A two-way analysis of variance (ANOVA) was performed to determine the effects of diet, training, and time. The statistical analysis was performed using GraphPad Prism 8.0 (GraphPad, Ltd., La Jolla, CA, USA). When ANOVA indicated a significant effect, a Tukey’s post hoc test was performed to determine the significance of differences between means. Associations between variables were analyzed using Pearson’s correlation coefficient. Statistical significance was set at *p* < 0.05.

## 3. Results

### 3.1. Weight Change and Relative Tissue or Organ Weight

**Figure 1:**
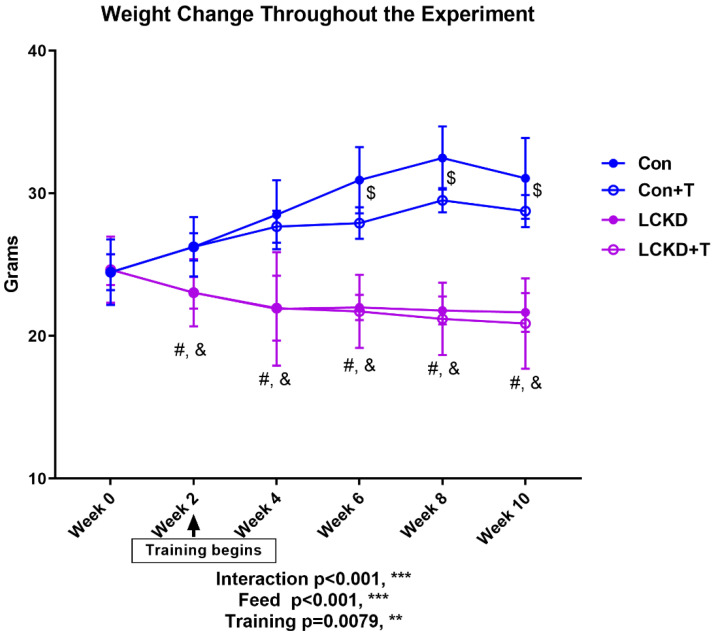
Weight change throughout the experiment. Con, Con + T, LCKD, and LCKD + T stand for chow diet, chow diet plus training, low-carbohydrate ketogenic diet, and low-carbohydrate ketogenic diet plus training, respectively. Feed indicates a main effect of diet, training indicates a main effect of treadmill exercise, and interaction indicates the interactive effect between feed and training. Interaction refers to the interactive effect between diet and training. ** *p* < 0.01 and *** *p* < 0.001. #, significant difference between Con and LCKD; &, significant difference between Con + T and LCKD + T; $, significant difference between Con and Con + T. A two-way analysis of variance (ANOVA) was performed to determine the effects of diet, training, and interaction. When ANOVA indicated a significant effect, a Tukey’s post hoc test was performed to determine the significance of differences between means. Statistical significance was set at *p* < 0.05. Data are presented as the mean ± standard deviation (SD). *n* = 8 for each group.

As shown in Figure 1, the average weight of mice in each group varied drastically. After training began at Week 2, significant differences in weight were observed between mice of the Con group and LCKD group. At the end of Week 6, a significant difference was observed between Con and Con + T mice. Throughout the whole study, no significant difference was observed between LCKD and LCKD + T.

**Figure 2:**
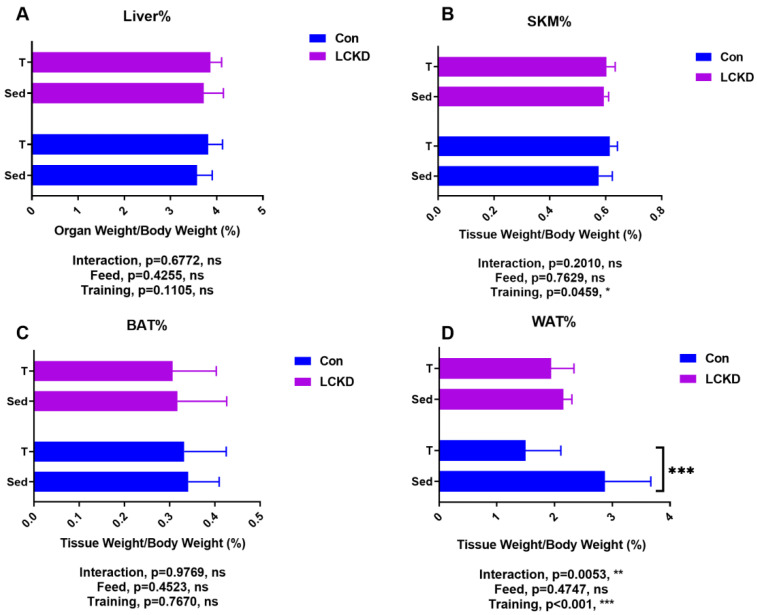
Tissues and organ weight/body weight ratio changes. (**A**) Relative ratio between whole liver and body weight; (**B**) relative ratio between skeletal muscle and body weight; (**C**) relative ratio between brown adipose tissue and body weight; (**D**) relative ratio between epididymal adipose tissue and body weight. SKM, skeletal muscle; WAT, white adipose tissue, epididymal adipose tissue; BAT, brown adipose tissue. % signifies the ratio between the indicated organ or tissue and the final weight. Con, Con + T, LCKD, and LCKD + T stand for chow diet, chow diet plus training, low-carbohydrate ketogenic diet, and low-carbohydrate ketogenic diet plus training, respectively. Feed indicates a main effect of diet, training indicates a main effect of treadmill exercise, and interaction indicates the interactive effect between feed and training. ns, no significance observed. * *p* < 0.05, ** *p* < 0.01, and *** *p* < 0.001. A two-way analysis of variance (ANOVA) was performed to determine the effects of diet, training, and interaction. When ANOVA indicated a significant effect, a Tukey’s post hoc test was performed to determine the significance of differences between means. Statistical significance was set at *p* < 0.05. Data are presented as the mean ± standard deviation (SD). *n* = 8 for each group.

As shown in Figure 2, the tissue/organ ratio of liver weight (Figure 2A) or brown adipose tissue weight (Figure 2C) to body weight did not significantly change due to training or diet. A muscle fascicle from the left hind leg containing the gastrocnemius, soleus, and plantaris muscles was also weighed. Training increased the skeletal muscle/body weight ratio, while diet had no effect (Figure 2B). Training also had a significant effect on the WAT (Figure 2D; WAT indicates epididymal adipose tissue) of mice, as WAT from training group mice weighed less than that from the non-training group mice. However, according to our results, this lowering effect only occurred in animals on the control diet. In the LCKD group, training did not reduce WAT weight.

### 3.2. Grip Power and Maximal Exercise Capacity Test

**Figure 3:**
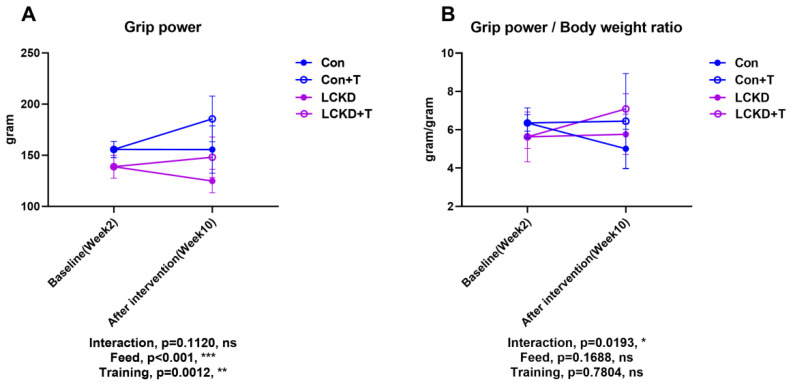
Forelimb grip power. (**A**) Absolute grip power and (**B**) grip power corrected by body weight. Con, Con + T, LCKD, and LCKD + T stand for chow diet, chow diet plus training, low-carbohydrate ketogenic diet, and low-carbohydrate ketogenic diet plus training, respectively. Feed indicates a main effect of diet, training indicates a main effect of treadmill exercise, and interaction indicates the interactive effect between feed and training. ns, no significance observed. * *p* < 0.05, ** *p* < 0.01, and *** *p* < 0.001. A two-way analysis of variance (ANOVA) was performed to determine the effects of diet, training, and interaction. When ANOVA indicated a significant effect, a Tukey’s post hoc test was performed to determine the significance of differences between means. Statistical significance was set at *p* < 0.05. Data are presented as the mean ± standard deviation (SD). *n* = 8 for each group.

To evaluate the effects of diet and training on muscle strength, grip power was assessed. Grip power was measured at two time points: at the beginning of Week 2, and again at the end of Week 10, after the training intervention finished. As shown in Figure 3A, training had a positive effect on grip-power performance. In fact, at the end of the experiment, a trend of increase in absolute grip power was observed between the Con and Con + T mice, as well as between the LCKD and LCKD + T mice. Taking the weight difference between the groups into consideration, we also corrected grip power based on body weight. As shown in Figure 3B, after the correction, while a decreasing trend was observed between Con mice at baseline and after intervention, a trend of increase was observed between the baseline LCKD and LCKD + T after intervention.

**Figure 4:**
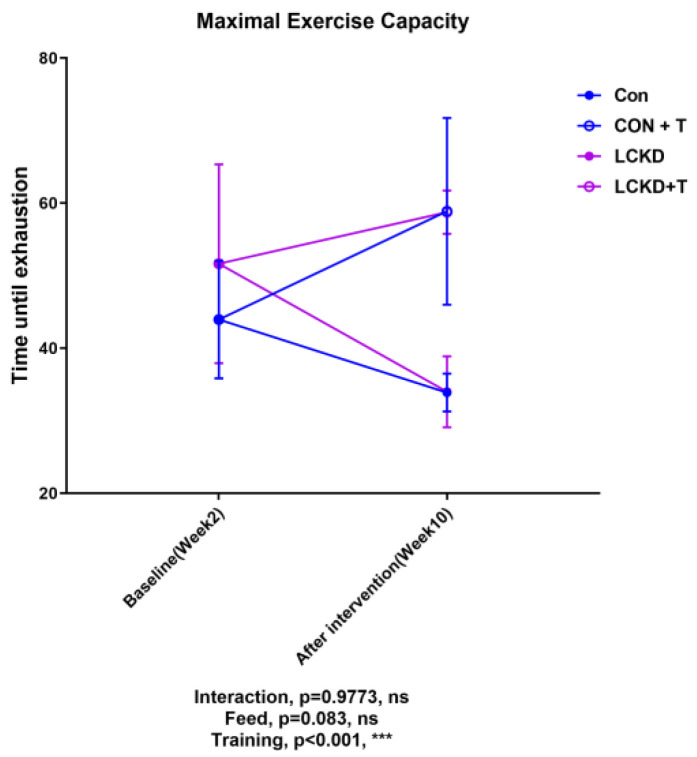
Maximal exercise capacity. Interaction refers to the interactive effect between diet and training. Con, Con + T, LCKD, and LCKD + T stand for chow diet, chow diet plus training, low-carbohydrate ketogenic diet, and low-carbohydrate ketogenic diet plus training, respectively. Feed indicates a main effect of diet, training indicates a main effect of treadmill exercise, and interaction indicates the interactive effect between feed and training. ns, no significance observed. *** *p* < 0.001. A two-way analysis of variance (ANOVA) was performed to determine the effects of diet, training, and interaction. When ANOVA indicated a significant effect, a Tukey’s post hoc test was performed to determine the significance of differences between means. Statistical significance was set at *p* < 0.05. Data are presented as the mean ± standard deviation (SD). *n* = 8 for each group.

As shown in Figure 4, at the end of the study, training enhanced maximal exercise capacity in both the Con and LCKD groups. Compared to baseline (Week 2), after a 2-month period of training, maximal exercise capacity was enhanced only in the Con group. The enhancement of exercise capacity in the LCKD group (baseline compared to after training period) was not significant.

Taking the considerable change in body weight between groups into consideration, we also calculated the maximal workload (Table 3). The 8-week training period significantly enhanced maximal workload in both the Con and LCKD groups, with significant differences observed between Con and Con + T, as well between as LCKD and LCKD + T.

### 3.3. Analysis of Plasma Biochemical Indexes

We further measured several fundamental biochemical indexes, including the plasma levels of cholesterol, glucose, albumin, BUN, creatinine, UA, NEFA, TG, LDL, HDL, and β-hydroxybutyrate. As shown in Table 4, among measured markers, the 10-week LCKD caused a dramatic increase in the plasma β-hydroxybutyrate concentration and significantly reduced plasma glucose levels. Total cholesterol, L-CHO, and H-CHO were all elevated due to the high fat content of the LCKD. Plasma BUN levels were significantly lowered in the LCKD group. Creatinine was lower in LCKD mice compared to Con mice, and was elevated in LCKD mice who underwent training compared to those that did not. The NEFA pool was also increased by the LCKD. Of note, the 8-week treadmill training regimen significantly lowered plasma TG concentrations, demonstrating the protective effects of exercise against hyperlipidemia. Albumin and UA levels were not affected by either the 10-week LCKD or the 8-week training regimen.

We used amylase, AST, CK, LDH, and lipase as muscle or organ damage markers. As shown in Table 5, plasma AST and lipase levels were significantly increased by the 10-week LCKD, while other markers were not altered by diet or training.

### 3.4. Plasma FGF21 and Testestorone Concentrations

**Figure 5:**
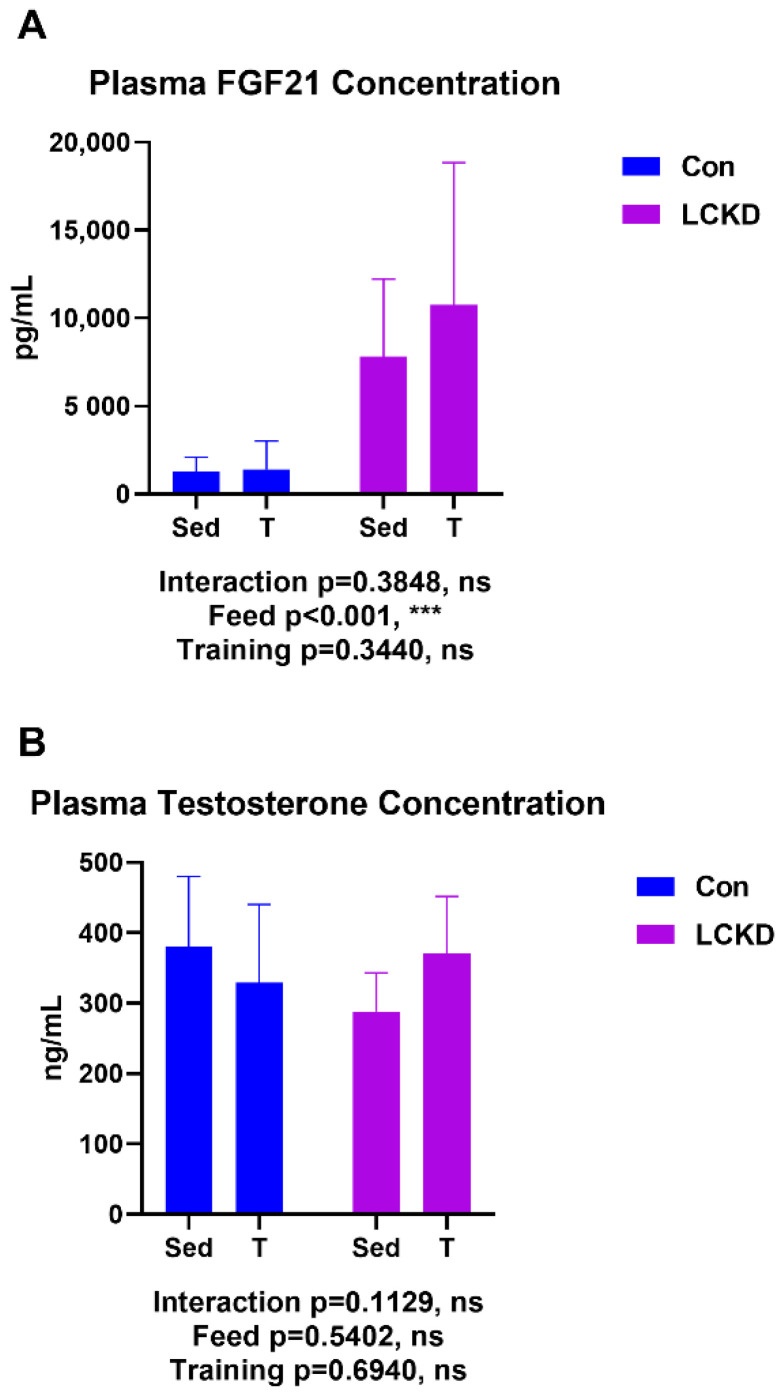
Plasma FGF21 and testosterone concentration. (**A**) Plasma FGF21 concentration; (**B**) Plasma testosterone concentration. FGF21, fibroblast growth factor 21; ns, no significance observed. Con, Con + T, LCKD, and LCKD + T stand for chow diet, chow diet plus training, low-carbohydrate ketogenic diet, and low-carbohydrate ketogenic diet plus training, respectively. Feed indicates a main effect of diet, training indicates a main effect of treadmill exercise, and interaction indicates the interactive effect between feed and training. *** *p* < 0.001. A two-way analysis of variance (ANOVA) was performed to determine the effects of diet, training, and interaction. Statistical significance was set at *p* < 0.05. Data are presented as the mean ± standard deviation (SD). *n* = 5–8 for each group.

As shown in Figure 5, plasma FGF21 levels were increased by the 10-week LCKD, while plasma testosterone was not altered by diet or training.

### 3.5. Muscle Glygocen Content

As shown in Table 6, among the groups, training exhibited a tendency to increase muscle glycogen content, but no significance was observed. The LCKD also did not affect muscle glycogen content.

### 3.6. Gene Expression in Tissues and Organs

We analyzed changes in the expression of relevant metabolism-associated genes in various tissues. As shown in Table 7, the 10-week LCKD decreased *Adiponectin* (anti-inflammation), *Atgl* (fatty acid mobilization), *F4/80* (inflammation), *Il-6* (inflammation), *Leptin* (energy metabolism)*,* and *Pgc1α* (energy metabolism) expression levels in WAT, while the expression of *Cd36* (fatty acid transportation), *Klotho* (FGF21 receptor), *Prdm16*, and *Pparγ* (browning of white adipose tissue) was not altered. The 8-week training regimen decreased *Leptin* expression in the control diet groups, but no synergistic effect of diet and training was observed in the low-carbohydrate ketogenic diet groups.

As shown in Table 8, we observed that the expression of *Ucp-1* in BAT was significantly upregulated by the 10-week LCKD. Training had no effect effects on *Ucp-1* expression, while other genes related to BAT thermogenesis function (*Cidea* and *Prdm16*) or inflammation (*Il6*) were not affected by neither factor. 

As shown in Table 9, in the gastrocnemius muscle, which represented fast-twitch, glycolytic muscle, the LCKD reduced glycogen synthesis, glycogenolysis, and glucose metabolism by downregulating *Gs* (glycogen synthesis), *Gp* (glycogen phosphatase), and *Hk2* (glycolysis). These changes were indicative of reduced glycometabolism resulting from the low carbohydrate intake during LCKD. *Mct1* (ketone body transportation) expression was upregulated by LCKD. The expression levels of *Acat1* (ketone body metabolism), *Hadh* (fatty acid oxidation), *Hbdh* (ketone body metabolism), *Oxct1* (ketone body metabolism), *Cd36* (fatty acid transportation), *Cox4* (mitochondrial respiration), *Cytochrome c* (mitochondrial respiration), *Sirt1* (mitochondrial respiration), and *Tfam* (mitochondrial respiration) were not significantly affected by either factor.

As shown in Table 10, in the soleus muscle, representative of slow-twitched oxidative muscle, we found that the expression of ketone body transporter *Mct1* (3.75 fold in LCKD and 2.67 fold in LCKD + T), fatty acid oxidation related *Cpt1α* (1.35 fold in LCKD and 1.29 fold in LCKD + T), *Hadh* (4.19 fold in LCKD and 4.28 fold in LCKD + T), and *Mcad* (3.75 fold in LCKD and 5.53 fold in LCKD + T) was upregulated. *Tfam* (mitochondrial respiration) and *Cox4* (mitochondrial respiration) expression was also increased (1.63 fold in LCKD and 1.38 fold in LCKD + T for *Tfam*, 2.00 fold in LCKD, and 2.51 fold in LCKD + T for *Cox4*) by LCKD, indicating an increase of mitochondrial biogenesis in the soleus muscle. *Il6*, which may induce lipolysis, was upregulated in the soleus muscle, but not significantly. The expression of key ketolysis genes *Acat1*, *Hbdh*, and *Oxct1* did not change significantly. 

## 4. Discussion

During LCKD feeding, the body enters a unique metabolic state that mimics the state of fasting. The exact mechanism through which an LCKD lowers weight is not completely understood, but possible mechanisms include: (1) appetite suppression [22]; (2) increased energy expenditure [23]; and (3) enhanced lipid metabolism and decreased triglyceride storage demand [19]. However, according to our results, LCKD mice consumed more calories, leaving (2) and (3) as possible underlying mechanisms.

In accordance with our previous results, a 10-week LCKD contributed to considerable weight loss [19]. Our current findings revealed that while the 8-week treadmill training regimen exhibited a tendency to reduce the average weight in mice on a chow diet, it did not have weight-lowering effects in LCKD-fed mice, which means that extra physical activity could not further contribute to weight loss when an LCKD was employed, or at least by a relatively short-term LCKD regimen combined with training. The current opinion of the effect of an LCKD on weight loss is controversial; two meta-analyses discussing this reached different conclusions: one showing that individuals assigned to an LCKD achieve a greater weight loss than those assigned to a low-fat diet [24]; while the other argued that weight loss was induced by either diet, a low-carbohydrate diet, or an isoenergetic balanced diet [25]. However, the above two studies did not involve training. Further studies are encouraged to find whether a longer period of LCKD and training could contribute to further weight loss, especially in lean subjects, since most of the previous studies were conducted in obese individuals. A similar pattern was observed in WAT weight change. Training significantly reduced WAT weight in the chow diet group, indicating that the training intensity (1 h/day, 5 days/week, at a fixed speed of 22.5 m/s) was sufficient to reduce body fat in a mouse model. Interestingly, training did not reduce fat weight in the LCKD group. While ketogenic diets have shown promising weight-control effects in various random controlled trials, few studies have been conducted in adults with a normal weight [26,27,28]. Our results indicated that a ketogenic diet may not induce further weight loss or body fat loss in such individuals.

Some researchers have suggested that due to the low protein content of the KD, loss of muscle volume may be a concern [29]. However, our results indicated that the relative muscle mass was not lower in LCKD-fed mice compared to controls. In addition to muscle weight, we evaluated muscle strength by employing a grip test. While the absolute grip strength decreased when corrected for body weight, an LCKD did not reduce grip strength. In general, exercise capacity includes endurance and explosive force capacity. The grip-power test evaluates the explosive force. Although muscle volume may not be influenced, the output rate of explosive power (mainly determined by fast-twitch muscle, utilizing glycometabolism as the energy source) may be compromised due to a deactivated and insufficient glycometabolic capacity [30]. The low average weight of individuals in the LCKD and LCKD + T groups may be another reason absolute grip power was lowered in the indicated groups. Although several studies reported that short-term LCKD or nutritional ketosis had no detrimental effect on power or strength in sports, such as artistic gymnastics and tactical athletes [31,32], other works suggested a detrimental effect, as observed in off-road cycling [33]. Our results indicated that an LCKD might be a preferable dietary approach for athletes that require output strength while maintaining a low body weight profile (e.g., in boxing, judo, etc.). Future studies should take this complicated effect of a long-term LCKD into consideration when evaluating its effects on athletes.

To evaluate whether a 10-week LCKD and/or an 8-week treadmill training enhanced maximal exercise capacity, we employed a graded treadmill exercise capacity test. According to previous studies, an 8-week ketogenic diet induced favorable changes in ketolytic gene expression and fatty acid oxidation gene expression, thus enhancing exercise performance in a mouse model [19]. Chronic endurance training has been confirmed to enhance fatty acid oxidation capacity. However, in our present study, the effects of diet and exercise were not additive. We employed a high-intensity evaluation system in the present study (uphill running with a maximal incline of 15 and a maximal speed of 42.5 m/s). However, when training was combined with a LCKD, the outcome was not improved compared with chow diet plus training. While an LCKD may favor lipid metabolism, high-intensity exercise requires high-output glycometabolism [34]. During low-intensity exercise, e.g., at 25% VO_2max_, enhanced lipolysis is observed in peripheral adipocytes. However, regardless of exercise intensity, lipolysis in the peripheral adipose tissue remains stable, while glycometabolism plays a pivotal role during high-intensity exercise [34]. Taking these observations into consideration, we further analyzed the changes in plasma biochemistry indexes, as well as changes in gene expression in various tissues, in order to determine to what extent LCKD affects the lipid pool and lipid metabolic capacity.

Among evaluated biomarkers, creatinine is released into blood at a constant rate depending on muscle mass. Mice in the LCKD group had lower creatinine levels compared to those in the other groups, indicative of reduced protein metabolism due to the low protein content of the diet, which is consistent with our previous results [18,19]. Plasma BUN levels were also significantly lower in the LCKD group, which may be another consequence of the low protein content of the LCKD.

AST was increased by an LCKD. AST is utilized as a key marker for the evaluation of hepatic damage. Previous studies reported that long-term high-fat diets, including the LCKD, might result in unfavorable alternations of hepatic function. Research has shown that the LCKD burdens hepatic function by aggravating endoplasmic reticulum stress and hepatocyte apoptosis [35,36]. In previous studies, an LCKD initially induced mild oxidative stress in the liver, thus activating the nuclear factor E2 related-factor 2 transcription factor [36,37]. It should be noted that the enhanced anti-oxidative reaction is a consequence of an LCKD, meant to counteract oxidative stress. Further, the use of antioxidants is recommended while on an LCKD regimen [38]. An LCKD improved plasma lipase levels, which may reflect an enhanced fat utilization capacity, since carbohydrate intake was restricted in both the LCKD and LCKD + T group. Amylase, a pancreatic damage marker; CK, a marker of muscle damage; and LDH, which is employed as a cardiac damage marker, were not affected by LCKD or by training. To summarize, an LCKD induced a unique metabolic status, characterized by an increased lipid pool and enhanced lipid oxidation capacity, and causing stress to hepatocytes. Further, the combination of an LCKD and training had no additive effects.

FGF21 is reported to induce thirst, as well as to improve whole-body energy metabolism, thus contributing to weight loss [39]. Both the LCKD and acute exercise are known to increase FGF21 concentration in the circulation [15,40]. In the present study, we demonstrated that while the LCKD elevated plasma FGF21, long-term training did not significantly affect FGF21 in the circulation. The enhanced level of FGF21 in the circulation may, to some extent, explain why the LCKD induced dramatic weight loss.

Exercise increases testosterone levels, while obesity induced by a high-fat diet may decrease testosterone, and supplementation of testosterone may contribute to maintaining muscle mass [16]. Our results showed that the 2-month training regimen or a 10-week LCKD had no significant effect on plasma testosterone levels. Therefore, training plus an LCKD may not affect testosterone-related muscle hypertrophy.

Due to the low carbohydrate content of the LCKD, we considered that the glycogen storage in muscle tissue may be reduced. Glycogen storage in LCKD-fed mice was not significantly decreased. Further, training increased muscle glycogen content. We also demonstrated that glycogen content was not reduced due to the low carbohydrate content in the LCKD. Further, training only exhibited a tendency to increase muscle glycogen content, reflecting the glycogen-loading effect. However, no significant difference was observed.

During exercise, the crosstalk between adipose tissue and muscle tissue is crucial [41]. Adiponectin, an adipokine secreted from the adipose tissue, regulates glucose and fatty acid metabolism directly, and via insulin-sensitizing effects, exhibits anti-inflammatory properties in the muscle, while levels of circulating adiponectin were reported to be decreased in obese individuals, and a negative correlation between adiponectin and circulating lipid profile was observed [42]. In the current study, the lipid pool in plasma increased as a result of the decrease in weight in LCKD mice. The expression level of adiponectin in the adipose tissue decreased. These results are rather complex when taking the other results into consideration. An LCKD may reduce adipose inflammation by suppressing immune-cell infiltration (as indicated by *F480* expression, employed as a marker for macrophage infiltration) and cytokine production (as indicated by *Il6* expression, employed as a marker for inflammation), while also downregulating adiponectin and thus reducing its metabolic effects. A 3–4-week LCKD was reported to alleviate inflammation in juvenile and adult rat models, while lipid accumulation was reported to downregulate *Atgl* and *Pgc1α* expression in *Drosophia* [43,44]. In agreement with previous studies, our work indicated that regular training may ameliorate inflammation in adipose tissue by downregulating *Il6* expression [45].

*Ucp-1*, also known as thermogenin, plays important roles in metabolic and energy balance, inducing non-shivering thermogenesis. Further, increasing the expression of *Ucp-1* may induce weight loss and be employed to treat obesity [46]. An LCKD usually induces weight loss, but the underlying mechanism has not been extensively explored. Our results revealed that an LCKD may induce weight loss by upregulating the expression of *Ucp-1* in BAT. Nakao et al. reported that an LCKD induced *Slc25a25*, an ATP carrier in the inner mitochondria membrane that is partially responsible for thermogenesis in muscle [47]. Considering this together with our current results, LCKD may induce weight loss through joint thermal regulation in both muscle tissue and BAT.

In the current work, we demonstrated that the glycogen content in the muscle tissue was not affected by the LCKD. We also hypothesized that the reason for the reduction in absolute grip power might be a deactivated glycometabolism system in glycolytic fast-twitch muscle. In slow-twitch oxidative muscle, ketone body transporter *Mct1*, as well as fatty acid oxidation-related genes *Cpt1α*, *Hadh*, and *Mcad*, were all upregulated, which was in agreement with previous studies [19]. *Tfam* and *Cox4* (mitochondrial respiration) were also upregulated by the LCKD, indicative of an increase in mitochondrial biogenesis in the soleus muscle and an enhanced capacity for KB transport and fatty acid oxidation. These results indicated that the fatty acid oxidation capacity in slow-twitch oxidative muscle was improved by the 10-week LCKD, which is in line with our previous results [19]. However, in our past works, we employed a low-intensity exhaustive exercise evaluation model, and an 8-week LCKD enhanced exercise capacity [18,19]. In the present study, even though fatty acid transport and oxidation genes were significantly upregulated, a 10-week LCKD did not improve the time to exhaustion in the maximal exercise capacity test when compared to the control diet. Therefore, an LCKD, which favors fatty acid oxidation, may not be useful for sports that require high-intensity energy output and vigorous glucose supply.

In a recent study, researchers reported that the response of liver metabolic pathways to the KD and exercise were not additive, although an LCKD alone did induce alternations as oxidative rates to catabolize lipids and ketogenic amino acids were enhanced; these effects, which may improve the energy metabolism and thus favor endurance and performance, were not further enhanced while combined with treadmill training [48]. We observed the same pattern in the muscle tissue, as the enhancing effects of the LCKD on lipid metabolism were not further improved by the addition of training.

The limitation of the present study is that only a maximal exercise capacity test was employed, while the activated lipid metabolic system exhibits potential to favor the low- to moderate-intensity exercise, further studies are encouraged to examine the synergistic effects of an LCKD and training using a different evaluating model. Another limitation is the absence of measuring baseline grip power before the feeding intervention started. Although after correction, the relative grip power was not decreased during LCKD feeding, the mechanisms remain unknown. IGF-1, which may be a key factor in maintaining muscle mass and strength, may be involved in the above process, while genes that involve in protein synthesis and breakdown that were not measured in the present study may be also adjusted by an LCKD, and further study is encouraged.

The major finding of the present study is that an LCKD enhanced the fatty acid oxidation capacity in oxidative muscle tissue, but this capacity was not further improved with training. The enhancement of fatty acid oxidation capacity did not, however, translate to an improvement in performance during highly intensive exercise. In addition, the muscle strength adjusted to weight was not compromised by the LCKD. Further studies are required to optimize the application of the LCKD to maximize its beneficial effects.

## 5. Conclusions

In the present study, we combined a 10-week LCKD with an 8-week treadmill training regimen to investigate whether this combination would favor exercise performance. The present results revealed that training enhanced maximal exercise capacity; though the LCKD had no practical effects to enhance the maximal exercise capacity, it was capable of enhancing the expression of genes that favor fatty acid oxidation in oxidative muscle, and thus may be promising for the improvement of endurance capacity during moderate-intensity exercise, but may not be an optimal choice for those partaking in high-intensity exercise.

## Figures and Tables

**Table 1:** Diet contents.

	Con	LCKD
Protein (%)	14.2	4.8
Fat (%)	10.1	93.2
Carbohydrate (%)	75.7	1.9
Kcal/g	3.6	7.3

**Table 2:** List of primers used for real-time PCR.

	Forward	Reverse
*18s*	CGGCTACCACATCCAAGGA	AGCTGGAATTACCGCGGC
*Adiponectin*	AGAGTCGTTGACGTTATCTGCATA	GGGCTCTGTGCTGCTCCATCT
*Atgl*	GAGCCCCGGGGTGGAACAAGAT	AAAAGGTGGTGGGCAGGAGTAAGG
*Cd36*	TGGCCTTACTTGGGATTGG	CCAGTGTATATGTAGGCTCATCCA
*F480*	CTTTGGCTATGGGCTTCCAGTC	GCAAGGAGGACAGAGTTTATCGTG
*Il-6*	AACGATGATGCACTTGCAGA	TGGTACTCCAGAAGACCAGAGG
*Klotho*	TGTTCTGCTGCGAGCTGTTAC	TACCGGACTCACGTACTGTTT
*Leptin*	GCTTTGGTCCTATCTGTCTTATGTT	CAATGGTCTTGATGAGGGTTTT
*Prdm16*	CCACCAGCGAGGACTTCA	GGAGGACTCTCGTAGCTCGAA
*Pgc1α*	GACTGGAGGAAGACTAAACGGCCA	GCCAGTCACAGGAGGCATCTTT
*Pparγ*	CCACCAGCGAGGACTTCAC	GGAGGACTCTCGTAGCTCGAA
*Cidea*	TCCTCGGCTGTCTCAATG	GGCTGCTCTTCTGTATCG
*Ucp-1*	TGGTTGGTTTTATTCGTGGT	AGGGTTTGTGGCTTCTTTTC
*Cox4*	TGGGAGTGTTGTGAAGAGTGA	GCAGTGAAGCCGATGAAGAAC
*Cs*	GCAGCCAAGAACTCATCCTG	TCTGGGCCTGCTCCTTAGGTA
*Cytochrome c*	CACGCTTTACCCTTCGTTCT	CTCATTTCCCTGCCATTCTC
*Gp*	TGGCAGAAGTGGTGAACAATGAC	CCGTGGAGATCTGCTCCGATA
*Gs*	ACTGCTTGGGCGTTATCTCTGTG	ATGCCCGCTCCATGCGTA
*Hadh*	ACTACATCAAAATGGGCTCTCAG	AGCAGAAATGGAATGCGGACC
*Hbdh*	AGTTTGGGGTCGAGGCTTTC	TGGTGGCCGCTATGAAGTTG
*Hk2*	CTGTCTACAAGAAACATCCCCATTT	CACCGCCGTCACCATAGC
*Mct1*	GCCTGAGCAAGTCAAGCTAG	TCAGACCTCGGATCCAGTAC
*Oxct1*	CCAAGGAAGTAAATGAAGATCTCCTA	ACGTGTATGTTACAAGAAATGGCTTACC
*Sirt1*	GCAACAGCATCTTGCCTGAT	GTGCTACTGGTCTCACTT
*Tfam*	TTCCCAAGACTTCATTTCATTGTC	GATGATTCGGCTCAGGGAAA
*Acat1*	CCGAGACAACTACCCAAGGA	CACACACAGGACCAGGACAC
*Cpt1α*	CCAGGCTACAGTGGGACATT	GAACTTGCCCATGTCCTTGT
*Mcad*	GCTCGTGAGCACATTGAAAA	CATTGTCCAAAAGCCAAACC

Note: 18s, 18s ribosomal RNA; Atgl, adipose triglyceride lipase; Cd36, cluster of differentiation 36; Il-6, interleukin-6; Prdm16, PR domain containing 16; Pgc1α, peroxisome proliferator activated receptor gamma coactivator-1alpha; Pparγ, peroxisome proliferatoractivated receptor gamma; Cidea, cell death inducing DNA fragmentation factor-alpha like effector A; Ucp-1, mitochondrial uncoupling protein 1; Cox4, cytochrome c oxidase polypeptide IV; Cs, citrate synthase; Gp, glycogen phosphatase; Gs, glycogen synthase; Hadh; hydroxyacyl-coA dehydrogenase; Hbdh, 3-hydroxybutyrate dehydrogenase; Hk2, hexokinase 2; Mct1, monocarboxylate transporter 1; Oxct1, 3-oxoacid CoA-transferase 1; Sirt1, sirtuin 1; Tfam, mitochondrial transcription factor A; Acat1, acetyl-coenzyme A acetyltransferase 1; Cpt, carnitine palmitoyltransferase; Mcad, medium chain acyl-CoA dehydrogenase.

**Table 3:** Maximal exercise workload after training intervention.

	Con	Con + T	LCKD	LCKD + T	Significance		
					Training	Feed	Interaction
MW *	19,641 ± 1345 ^bc^	41,378 ± 1269 ^ad^	11,680 ± 512 ^ad^	27,385 ± 3282 ^bd^	***	***	***

Note: * MW, Maximal workload measured at the end of Week 10. Maximal workload was defined as total climbed vertical distance × weight. Con, Con + T, LCKD, and LCKD + T stand for chow diet, chow diet plus training, low-carbohydrate ketogenic diet, and low-carbohydrate ketogenic diet plus training, respectively. Feed indicates a main effect of diet, training indicates a main effect of treadmill exercise, and interaction indicates the interactive effect between feed and training. *** *p* < 0.001. ^a^, significantly different from Con; ^b^, significantly different from Con + T; ^c^, significantly different from LCKD; ^d^, significantly different from LCKD + T. A two-way analysis of variance (ANOVA) was performed to determine the effects of diet, training, and interaction. When ANOVA indicated a significant effect, a Tukey’s post hoc test was performed to determine the significance of differences between means. Statistical significance was set at *p* < 0.05. Data are presented as the mean ± standard deviation (SD). *n* = 8 for each group.

**Table 4:** The effects of LCKD feeding and/or training on plasma biochemical markers.

	Con	Con + T	LCKD	LCKD + T	Significance		
					Training	Feed	Interaction
β-hydroxybutyrate, mmol/L	0.40 ± 0.42	0.31 ± 0.35	4.48 ± 1.91	5.00 ± 3.22	n. s.	***	n. s.
Albumin, mg/dL	2.55 ± 0.23	2.43 ± 0.19	2.33 ± 0.65	2.21 ± 0.27	n. s.	n. s.	n. s.
BUN, mg/dL	23.44 ± 5.02	25.08 ± 3.50	16.08 ± 4.40	15.45 ± 1.81	n. s.	***	n. s.
Creatinine, mg/dL	0.10 ± 0.03 ^c^	0.12 ± 0.02	0.07 ± 0.03 ^ad^	0.12 ± 0.02 ^c^	***	*	**
Glucose, mg/dL	201.4 ± 58.67	212.3 ± 33.78	135.8 ± 31.74	123.8 ± 33.17	n. s.	***	n. s.
T-CHO, mg/dL	100.9 ± 10.13	93.38 ± 8.40	135.8 ± 31.74	144.0 ± 31.34	n. s.	***	n. s.
L-CHO, mg/dL	13.88 ± 3.56	15.00 ± 4.81	23.63 ± 4.66	24.00 ± 7.52	n. s.	***	n. s.
H-CHO, mg/dL	76.13 ± 8.17	69.75 ± 6.94	107.3 ± 28.41	111.0 ± 25.25	n. s.	***	n. s.
NEFA, μEq/L	2.85 ± 0.31	2.64 ± 0.19	2.68 ± 0.52 ^d^	3.11 ± 0.37 ^c^	n. s.	n. s.	*
TG, mg/dL	20.63 ± 7.25	16.13 ± 7.85	21.75 ± 11.08	14.14 ± 4.81	*	n. s.	n. s.
UA, mg/dL	1.39 ± 0.51	1.39 ± 0.51	1.35 ± 0.89	1.35 ± 0.89	n. s.	n. s.	n. s.

Note: BUN, blood urea nitrogen; T-CHO, total cholesterol; L-CHO, low density lipoprotein cholesterol; H-CHO, high density lipoprotein cholesterol; NEFA, non-esterified fatty acid; TG, triglyceride; UA, uric acid. Con, Con + T, LCKD, and LCKD + T stand for chow diet, chow diet plus training, low-carbohydrate ketogenic diet, and low-carbohydrate ketogenic diet plus training, respectively. Feed indicates a main effect of diet, training indicates a main effect of treadmill exercise, and interaction indicates the interactive effect between feed and training. n. s., no significance was observed. * *p* < 0.05, ** *p* < 0.01, and *** *p* < 0.001. ^a^, significantly different from Con; ^c^, significantly different from LCKD; ^d^, significantly different from LCKD + T. A two-way analysis of variance (ANOVA) was performed to determine the effects of diet, training, and interaction. When ANOVA indicated a significant effect, a Tukey’s post hoc test was performed to determine the significance of differences between means. Statistical significance was set at *p* < 0.05. Data are presented as the mean ± standard deviation (SD). *n* = 8 for each group.

**Table 5:** The effect of LCKD feeding and/or training on plasma muscle/organ damage indicators.

	Con	Con + T	LCKD	LCKD + T	Significance		
					Training	Feed	Interaction
Amylase, IL/L	1755 ± 252	1623 ± 220	1525 ± 341	1664 ± 155	n. s.	n. s.	n. s.
AST, IU/L	81 ± 47	69 ± 48	151 ± 86	152 ± 92	n. s.	**	n. s.
CK, IU/L	155 ± 130	136 ± 205	192 ± 121	228 ± 130	n. s.	n. s.	n. s.
LDH, IU/L	421 ± 290	881 ± 299	536 ± 463	548 ± 224	n. s.	n. s.	n. s.
Lipase, IU/L	40 ± 7	46 ± 6	66 ± 15	74 ± 13	n. s.	***	n. s.

Note: AST, aspartate transaminase; CK, creatinine kinase; LDH, lactate dehydrogenase. Con, Con + T, LCKD, and LCKD + T stand for chow diet, chow diet plus training, low-carbohydrate ketogenic diet, and low-carbohydrate ketogenic diet plus training, respectively. Feed indicates a main effect of diet, training indicates a main effect of treadmill exercise, and interaction indicates the interactive effect between feed and training. n. s., no significance was observed. ** *p* < 0.01 and *** *p* < 0.001. A two-way analysis of variance (ANOVA) was performed to determine the effects of diet, training, and interaction. When ANOVA indicated a significant effect, a Tukey’s post hoc test was performed to determine the significance of differences between means. Statistical significance was set at *p* < 0.05. Data are presented as the mean ± standard deviation (SD). *n* = 8 for each group.

**Table 6:** The effect of LCKD feeding and/or training on muscle glycogen content.

	Con	Con + T	LCKD	LCKD + T	Significance		
					Training	Feed	Interaction
Muscle glycogen, μg/g protein	26.3 ± 9	35.2 ± 2.5	27.3 ± 1.17	32.4 ± 0.12	n. s.	n. s.	n. s.

Note: Con, Con + T, LCKD, and LCKD + T stand for chow diet, chow diet plus training, low-carbohydrate ketogenic diet, and low-carbohydrate ketogenic diet plus training, respectively. Feed indicates a main effect of diet, training indicates a main effect of treadmill exercise, and interaction indicates the interactive effect between feed and training. n. s., no significance was observed. A two-way analysis of variance (ANOVA) was performed to determine the effects of diet, training, and interaction. Statistical significance was set at *p* < 0.05. Data are presented as the mean ± standard deviation (SD). *n* = 3–5 for each group.

**Table 7:** The effect of LCKD feeding and/or training on WAT gene expression.

	Con	Con + T	LCKD	LCKD + T	Significance		
					Training	Feed	Interaction
*Adiponectin* (Anti-inflammation)	1.00 ± 0.46	1.01 ± 0.29	0.44 ± 0.24	0.52 ± 0.14	n. s.	***	n. s.
*Atgl* (Fatty acid mobilization)	1.00 ± 0.40	0.95 ± 0.43	0.30 ± 0.12	0.30 ± 0.14	n. s.	*	n. s.
*Cd36* (Fatty acid transportation)	1.00 ± 0.36	0.90 ± 0.29	0.51 ± 0.37	0.45 ± 0.32	n. s.	n. s.	n. s.
*F4/80* (Inflammation)	1.00 ± 0.30	0.91 ± 0.20	0.50 ± 0.24	0.55 ± 0.25	n. s.	***	n. s.
*Il-6* (Inflammation)	1.00 ± 0.51	0.53 ± 0.19	0.26 ± 0.14	0.21 ± 0.61	**	***	n. s.
*Klotho* (FGF21 receptor)	1.00 ± 0.34	1.05 ± 0.50	0.95 ± 0.64	0.91 ± 0.55	n. s.	n. s.	n. s.
*Leptin* (Energy metabolism)	1.00 ± 0.43 ^bc^	0.49 ± 0.34 ^a^	0.43 ± 0.39 ^a^	0.50 ± 0.23	n. s.	*	*
*Prdm16* (Browning of WAT)	1.00 ± 0.29	0.88 ± 0.22	1.07 ± 1.23	0.75 ± 0.61	n. s.	n. s.	n. s.
*Pgc1α* (Browning of WAT)	1.00 ± 0.67	1.23 ± 0.68	0.63 ± 0.32	0.63 ± 0.36	n. s.	*	n. s.
*Pparγ* (Browning of WAT)	1.00 ± 0.51	1.23 ± 0.54	0.69 ± 0.44	0.90 ± 0.63	n. s.	n. s.	n. s.

Note: WAT, wat adipose tissue. Data (fold changes) are presented as the mean ± SD. Con, Con + T, LCKD, and LCKD + T stand for chow diet, chow diet plus training, low-carbohydrate ketogenic diet, and low carbohydrate ketogenic diet plus training, respectively. Feed indicates a main effect of diet, training indicates a main effect of treadmill exercise, and interaction indicates the interactive effect between feed and training. n. s., no significance was observed. * *p* < 0.05, ** *p* < 0.01, and *** *p* < 0.001. ^a^, significantly different from Con; ^b^, significantly different from Con + T; ^c^, significantly different from LCKD. A two-way analysis of variance (ANOVA) was performed to determine the effects of diet, training, and interaction. Statistical significance was set at *p* < 0.05 *n* = 4–8 for each group.

**Table 8:** Effect of LCKD feeding and/or training on BAT gene expression.

	Con	Con + T	LCKD	LCKD + T	Significance		
					Training	Feed	Interaction
*Cidea* (Thermogenesis)	1.00 ± 0.15	1.20 ± 0.07	1.08 ± 0.89	0.81 ± 0.12	n. s.	n. s.	n. s.
*Il-6* (Inflammation)	1.00 ± 0.51	1.45 ± 0.55	1.89 ± 2.65	1.32 ± 0.42	n. s.	n. s.	n. s.
*Prdm16* (Thermogenesis)	1.00 ± 0.28	1.15 ± 0.34	0.83 ± 0.61	0.59 ± 0.08	n. s.	n. s.	n. s.
*Ucp1* (Thermogenesis)	1.00 ± 0.24	1.60 ± 0.54	2.42 ± 1.82	1.85 ± 0.43	n. s.	*	n. s.

Note: Data (fold changes) are presented as the mean ± SD. Con, Con + T, LCKD, and LCKD + T stand for chow diet, chow diet plus training, low-carbohydrate ketogenic diet, and low-carbohydrate ketogenic diet plus training, respectively. Feed indicates a main effect of diet, training indicates a main effect of treadmill exercise, and interaction indicates the interactive effect between feed and training. n. s., no significance was observed. * *p* < 0.05. A two-way analysis of variance (ANOVA) was performed to determine the effects of diet, training, and interaction. Statistical significance was set at *p* < 0.05. *n* = 4–8 for each group.

**Table 9:** The effect of LCKD feeding and/or training on gastrocnemius muscle gene expression.

	Con	Con + T	LCKD	LCKD + T	Significance		
					Training	Feed	Interaction
*Acat1* (Ketone body metabolism)	1.00 ± 0.46	1.05 ± 0.44	1.30 ± 1.45	0.75 ± 0.23	n. s.	n. s.	n. s.
*Cd36* (Fatty acid transportation)	1.00 ± 0.28	0.99 ± 0.29	0.70 ± 0.32	0.75 ± 0.26	n. s.	n. s.	n. s.
*Cox4* (Mitochondrial respiration)	1.00 ± 0.30	0.97 ± 0.28	0.65 ± 0.29	0.79 ± 0.14	n. s.	n. s.	n. s.
*Cs (Citrate synthase)* (Mitochondrial respiration)	1.00 ± 0.23	1.19 ± 0.43	0.73 ± 0.32	1.02 ± 0.11	n. s.	n. s.	n. s.
*Cytochrome c* (Mitochondrial respiration)	1.00 ± 0.28	1.12 ± 0.44	0.90 ± 0.55	1.08 ± 0.28	n. s.	n. s.	n. s.
*Gp* (Glycogen phosphatase)	1.00 ± 0.25	1.21 ± 0.68	0.71 ± 0.35	0.52 ± 0.19	n. s.	*	n. s.
*Gs* (Glycogen synthase)	1.00 ± 0.40	0.83 ± 0.40	0.66 ± 0.53	0.65 ± 0.49	n. s.	*	n. s.
*Hadh* (Fatty acid oxidation)	1.00 ± 0.27	0.96 ± 0.37	1.27 ± 0.57	1.22 ± 0.36	n. s.	n. s.	n. s.
*Hbdh* (Ketone body metabolism)	1.00 ± 0.45	0.95 ± 0.44	0.55 ± 0.35	0.31 ± 0.18	n. s.	n. s.	n. s.
*Hk2* (Glycolysis)	1.00 ± 0.11	1.16 ± 0.37	0.61 ± 0.28	0.60 ± 0.12	n. s.	*	n. s.
*Mct1* (Ketone body transportation)	1.00 ± 0.22	0.93 ± 0.43	1.78 ± 0.84	1.34 ± 0.39	n. s.	*	n. s.
*Oxct1* (Ketone body metabolism)	1.00 ± 1.10	0.53 ± 0.30	0.35 ± 0.22	0.34 ± 0.08	n. s.	n. s.	n. s.
*Sirt1* (Mitochondrial respiration)	1.00 ± 0.39	0.79 ± 0.18	0.65 ± 0.15	0.62 ± 0.14	n. s.	n. s.	n. s.
*Tfam* (Mitochondrial respiration)	1.00 ± 0.27	1.09 ± 0.42	0.88 ± 0.30	0.97 ± 0.24	n. s.	n. s.	n. s.

Note: Data (fold changes) are presented as the mean ± SD. Con, Con + T, LCKD, and LCKD + T stand for chow diet, chow diet plus training, low-carbohydrate ketogenic diet, and low-carbohydrate ketogenic diet plus training, respectively. Feed indicates a main effect of diet, training indicates a main effect of treadmill exercise, and interaction indicates the interactive effect between feed and training. n. s., no significance was observed. * *p* < 0.05. A two-way analysis of variance (ANOVA) was performed to determine the effects of diet, training, and interaction. Statistical significance was set at *p* < 0.05. *n* = 4–8 for each group.

**Table 10:** The effect of LCKD and/or training on soleus muscle gene expression.

	Con	Con + T	LCKD	LCKD + T	Significance		
					Training	Feed	Interaction
*Acat1* (Ketone body metabolism)	1.00 ± 0.35	0.51 ± 0.16	0.70 ± 0.19	0.85 ± 0.28	n. s.	n. s.	n. s.
*Cox4* (Mitochondrial respiration)	1.00 ± 0.33	0.62 ± 0.17	2.00 ± 0.33	2.51 ± 0.70	n. s.	*	n. s.
*Cpt1α* (Fatty acid oxidation)	1.00 ± 0.23	0.61 ± 0.21	1.35 ± 0.34	1.29 ± 0.37 ^b^	n. s.	***	n. s.
*Hadh* (Fatty acid oxidation)	1.00 ± 0.59	0.67 ± 0.30	4.19 ± 0.90	4.28 ± 1.70	n. s.	***	n. s.
*Hbdh* (Ketone body metabolism)	1.00 ± 0.51	0.70 ± 0.25	0.64 ± 0.06	0.60 ± 0.19	n. s.	n. s.	n. s.
*Hk2* (Glycolysis)	1.00 ± 0.42	1.09 ± 0.13	0.93 ± 0.30	0.87 ± 0.27	n. s.	n. s.	n. s.
*Il-6* (Fatty acid mobilization)	1.00 ± 0.57	1.05 ± 0.57	1.64 ± 1.04	1.35 ± 0.48	n. s.	n. s.	n. s.
*Oxct1* (Ketone body metabolism)	1.00 ± 1.53	0.13 ± 0.06	0.34 ± 0.11	0.23 ± 0.06	n. s.	n. s.	n. s.
*Mcad* (Fatty acid oxidation)	1.00 ± 0.64	0.55 ± 0.31	3.75 ± 1.10	5.53 ± 2.75	n. s.	***	n. s.
*Mct1* (Ketone body transportation)	1.00 ± 0.85	1.32 ± 0.52	3.75 ± 1.30	2.67 ± 0.64	n. s.	***	n. s.
*Tfam* (Mitochondrial respiration)	1.00 ± 0.53	0.82 ± 0.26	1.63 ± 0.50	1.38 ± 0.63	n. s.	*	n. s.

Note: Data (fold changes) are presented as the mean ± standard deviation (SD). Con, Con + T, LCKD, and LCKD + T stand for chow diet, chow diet plus training, low-carbohydrate ketogenic diet, and low-carbohydrate ketogenic diet plus training, respectively. Feed indicates a main effect of diet, training indicates a main effect of treadmill exercise, and interaction indicates the interactive effect between feed and training. n. s., no significance was observed. * *p* < 0.05 and *** *p* < 0.001. ^b^, significantly different from Con + T. A two-way analysis of variance (ANOVA) was performed to determine the effects of diet, training, and interaction. Statistical significance was set at *p* < 0.05. *n* = 4–8 for each group.
